# Prevalence of Loneliness and Associated Factors among Community-Dwelling Older Adults in Indonesia: A Cross-Sectional Study

**DOI:** 10.3390/ijerph19084911

**Published:** 2022-04-18

**Authors:** Sri Susanty, Min-Huey Chung, Hsiao-Yean Chiu, Mei-Ju Chi, Sophia H. Hu, Chien-Lin Kuo, Yeu-Hui Chuang

**Affiliations:** 1 School of Nursing, College of Nursing, Taipei Medical University, 250 Wu-Xing St., Taipei 110, Taiwan; sri.susanty@uho.ac.id (S.S.); minhuey300@tmu.edu.tw (M.-H.C.); hychiu0315@tmu.edu.tw (H.-Y.C.); 2 Nursing Study Program, Faculty of Medicine, Universitas Halu Oleo, Tridharma Green Campus, H.E.A. Mokodompit St., Kendari 93232, Indonesia; 3 Department of Nursing, Shuang-Ho Hospital, Taipei Medical University, 291 Zhongzheng Rd., Zhonghe District, New Taipei City 235, Taiwan; 4 Research Center of Sleep Medicine, College of Medicine, Taipei Medical University, 250 Wu-Xing St., Xinyi District, Taipei 110, Taiwan; 5 Department of Nursing, Taipei Medical University Hospital, 252 Wu-Xing St., Xinyi District, Taipei 110, Taiwan; 6 School of Gerontology Health Management, College of Nursing, Taipei Medical University, 250 Wu-Xing St., Xinyi District, Taipei 110, Taiwan; mjchi@tmu.edu.tw; 7 Master Program in Long-Term Care, College of Nursing, Taipei Medical University, 250 Wu-Xing St., Xinyi District, Taipei 110, Taiwan; 8 Department of Nursing, College of Nursing, National Yang Ming Chiao Tung University, 155 Sec. 2, Linong St., Peitou District, Taipei 112, Taiwan; sophiahu@nycu.edu.tw; 9 Department of Allied Health Education & Digital Learning, National Taipei University of Nursing and Health Sciences, 365 Ming-Te Road, Peitou District, Taipei 112, Taiwan; chienlin@ntunhs.edu.tw; 10 Center for Nursing and Healthcare Research in Clinical Application, Wan Fang Hospital, Taipei Medical University, 111 Sec. 3, Xinglong Rd., Wenshan District, Taipei 116, Taiwan

**Keywords:** community, Indonesia, loneliness, older adults, prevalence

## Abstract

Loneliness has become one of the most common psychological problems experienced by older adults. Previous studies have indicated that loneliness is correlated with poor physical and psychological health outcomes; therefore, it is important to pay attention to people experiencing loneliness. However, there is a lack of information regarding the prevalence of loneliness, and its associated factors, among community-dwelling older adults in Indonesia, which this study aimed to understand. This study used a cross-sectional, descriptive, and correlational research design. Stratified random sampling was applied to 1360 participants, aged ≥ 60 years, in 15 community health centers in Kendari City, Indonesia. The following questionnaires were used to collect data, including demographic and characteristic information, Short Portable Mental Status Questionnaire, Multidimensional Scale of Perceived Social Support, Geriatric Depression Scale Short Form, and a single-item loneliness question. The prevalence of loneliness among older adults was 64.0%. The multivariate logistic regression showed that older adults who were female, lived with family, had fewer children, had a poor health status, had a poor oral status, had more chronic diseases, had no hearing problems, had poor cognitive function, and had depression had a higher chance of feeling lonely. Loneliness is a serious health issue among the older population in Indonesia. The government, social workers, and healthcare professionals should pay immediate attention to this psychological problem. The study also suggests that appropriate strategies for the prevention of loneliness should be developed in the near future.

## 1. Introduction

Loneliness is a common mental health problem, especially in older adults, caused due to exposure to certain factors related to old age [1]. It is a subjective feeling of unpleasantness [2], sadness [3], emptiness, distress, suffering, isolation, lack of meaning, non-belongingness, or lack of companionship [4]. Some scholars believe that individual unmet social needs, either in quantity or quality of social relationships, might trigger people to suffer loneliness [5].

The prevalence of loneliness among community-dwelling older adults varies across countries. Based on national surveys, 7.68–9.88% of Swedish older adults (aged ≥ 70 years) had feelings of loneliness [6], and 11.6% of 2052 home-dwelling older persons (aged ≥ 65 years) often felt lonely in Norway [7]. In addition to European countries, studies in the USA also found that the prevalence of loneliness in the elderly population was 26.2% in Chinese immigrants, 43% in people aged ≥60 years in a nationally representative study, and 44% in elderly veterans [4,8,9]. Similarly, the prevalence of loneliness in older adults was also high, at 38.7% (moderate level of loneliness) and 16.9% (severe level of loneliness), in community-dwelling older adults in Nepal [10]. In China, Teh and Tey [11] found that 8% of the older population (aged ≥60 years) always or often felt lonely.

When loneliness becomes severe and prolonged, it can jeopardize an individual’s physical and psychological health. A systematic review showed that loneliness was significantly associated with heart disease, hypertension, stroke, and lung disease [12]. In addition to disease, loneliness affects other health outcomes, such as declining physical function [4], sleep fragmentation [13], and poor sleep quality [14]. Loneliness is also related to negative mental health, such as psychological distress [15], anxiety, depression [16,17], and impaired cognitive functioning and dementia [18]. Furthermore, loneliness significantly increases the possibility of committing suicide [19] and is associated with a higher risk of mortality [20].

Previous studies found that age, gender, marital status, education, income, poor self-reported health [21], religion [22], activities of daily living (ADL) [4], number of children, instrumental activities of daily living (IADL) [23], living status [24], oral health, teeth problems [25,26], visual problems, employment status [27,28], hearing problems [29], chronic disease [30], leisure activities [11], social support [25], depression [31], and cognitive function [32] were significantly related to loneliness in older people. Loneliness has negative effects on an individual’s physical and mental health, so it is important for healthcare professionals to understand the prevalence rates of loneliness and its related factors. However, the majority of Indonesian studies on loneliness have targeted the general population [33] or adolescents [34]. To the best of our knowledge, there have been few studies in Indonesia that have examined the prevalence of loneliness among older adults in the community. Therefore, the aim of this study was to understand the prevalence of loneliness and its associated factors among community-dwelling older adults in Indonesia. The hypothesis of this study was that there were associations between older adults’ demographics and characteristics and their feelings of loneliness.

## 2. Materials and Methods

### 2.1. Research Design 

A cross-sectional, descriptive, and correlational design was used in this study. This study is a part of a project titled “Psychological well-being among older adults who live in the community in Indonesia.”

### 2.2. Research Setting and Sample

This study was conducted across 15 community health centers (CHCs) in Kendari City, Southeast Sulawesi, Indonesia. Southeast Sulawesi is a province in Indonesia located in the southeastern part of the island of Sulawesi, and Kendari City is the capital of Southeast Sulawesi. There were 345,107 people in Kendari City in 2020, and approximately 21,236 (6.15%) older adults consisting of 50.4% males and 49.5% females [35]. Among these older adults, 16,652 people received services from CHCs during the period of data collection. The CHCs are technical implementation units, and their primary function is to serve as first-level healthcare providers. Both healthy and ill people can utilize CHC services [35].

Stratified random sampling was used, based on the proportion of older people in each CHC (30% in Baruga, 20% in Western Kandai, 15% in Kadia, 10% in Central Kendari, 8% in Wua-Wua, 5% in Kambu, 4% in Poasia, 3% in Mandonga, 2% in Puuwatu, and 1% in Abeli and Nambo). The inclusion criteria of this study were persons aged ≥ 60 years, who could speak Indonesian or the local language, and could communicate with others. The exclusion criteria were persons who had any psychiatric disorders, such as schizophrenia. The rationale for recruiting persons who were 60 years old and over was that older adults are defined as those aged 60 years and above in Indonesia. The sample size was calculated by G* Power 3.1 [36]. The parameters were alpha = 0.05, power = 0.8, effect size = 0.02 [37]; the number of predictors was 23, and the estimated sample size was 1124. After excluding 31 older adults based on the abovementioned criteria, we invited 1435 eligible older adults to participate in the study; however, 75 older adults refused to participate. Ultimately, 1360 participants joined the study. 

### 2.3. Data Collection Procedure

After obtaining institutional approval, eligible participants from each CHC were randomly selected using a computer. Selected participants were approached either during their visit to the CHC or via a home visit. The researchers explained the purpose of the study and the collection procedures, and verbal consent was obtained. Participants completed the questionnaires on their own. If they were illiterate, the researchers read the questions and helped them to write down the answers to the questionnaires. It took approximately 30–45 min per participant. Thereafter, the participants placed the questionnaires in a sealed box. The participants who returned the questionnaires to the sealed box indicated that they agreed to participate in the study. Data were collected from June 2019 to September 2019.

### 2.4. Instruments

The questionnaire consisted of five parts, including demographic and characteristic information, Short Portable Mental Status Questionnaire, Multidimensional Scale of Perceived Social Support, Geriatric Depression Scale Short Form, and a single-item loneliness question.

#### 2.4.1. Demographic and Characteristic Information

This included age, gender, educational level, religion, marital status, living status, number of children, previous employment, current job, income, health status, oral health status, chronic disease, teeth problems, vision problems, hearing problems, attending leisure activities (number of times/month), activities of daily living (ADLs), and instrumental ADLs (IADLs). The ten-item Barthel Index (BI) to measure ADLs, developed by Mahoney and Barthel [38], was used to assess individual independence. The Indonesian version of the BI was used in this study. The score ranged from 0 to 100. A higher score indicated greater independence of the participants. Cronbach’s alpha was 0.94 [39]. The eight-item Lawton IADL Scale was used to assess individual independent living skills in the community. The Indonesian version of the Lawton IADL scale was used in this study [40]. The total score ranged from 0 to 8, with higher values indicating greater independence. Cronbach’s alpha was 0.91 [41].

#### 2.4.2. The Short Portable Mental Status Questionnaire (SPMSQ)

The SPMSQ was developed by Pfeiffer [42]. The Indonesian version of the SPMSQ was used to measure the participants’ cognitive function. It includes 10 questions; 0 points are given for an incorrect answer and 1 point for a correct answer. The total scores range from 0 to 10. A higher score indicates greater intact cognitive functioning of participants. The test–retest reliability of the Indonesian version of the scale was 0.80 [43].

#### 2.4.3. Multidimensional Scale of Perceived Social Support (MSPSS)

The 12-item MSPSS was used to measure participants’ social support levels from three sources (family, friends, and significant others) [44]. Each source consisted of four items. The Indonesian version of the MSPSS developed by Winahyu et al. [45] was used in this study. A 7-point Likert scale ranging from “very strongly disagree” to “very strongly agree” was used to rate each item. The total score ranged from 12 to 84. A higher score indicated a higher level of support. The Cronbach’s alpha for the Indonesian version of the MSPSS was 0.85 [45].

#### 2.4.4. Geriatric Depression Scale Short Form (GDS-SF)

The 15-item GDS-SF was developed by Yesavage and Sheikh [46] to screen for depression in older adults. The Indonesian version of the GDS-SF was used in this study [47]. The GDS-SF consists of 15 questions with “yes” or “no” responses. The score ranges from 0 to 15, with a higher score indicating a higher degree of depression. Cronbach’s alpha for the Indonesian version of the GDS-SF was 0.80 [47].

#### 2.4.5. Loneliness Scale

A single-item question (“How often do you feel lonely?”) was used to determine whether the participants were experiencing loneliness [48]. Previous studies have used this single item to measure loneliness [6,33], and it has a significant correlation (r = 0.79, *p* < 0.001) with the UCLA Loneliness Scale [2]. There are four response categories: “always,” “sometimes,” “seldom,” and “never.” Answering “always” and “sometimes” scores 1 point, and answering “seldom” and “never” scores 0 points. One point indicated that a participant experienced loneliness.

### 2.5. Data Analysis

IBM Statistical Package for the Social Sciences (SPSS) for Mac version 26.0. (IBM Corp. Armonk, NY, USA) was used for data analysis. The mean, standard deviation (SD), frequency, and percentage were calculated to describe older adults’ demographic and characteristic information, cognitive function, social support, depression, and loneliness. Univariate logistic regression was used to determine the association between the independent variables and loneliness. Multivariate logistic regression was used to identify the predictors of loneliness. There were a few missing data in the current study, and the mean value of this particular variable was used to fill in the missing values. Statistical significance was set at *p* < 0.05.

## 3. Results

### 3.1. Demographic Characteristics of Study Participants

The mean age among the 1360 participants was 66.28 (SD = 6.39) years and ranged from 60 to 100 years. Five hundred and forty-nine (40.4%) were male and 811 (59.6%) were female. Most participants were literate (86.2%) and were Muslim (97.2%). The majority of older adults had partners (69.6%), lived with their family (93.8%), and had an average of 3.83 (SD = 2.34) children. Over half of the older adults had been employed before (54.0%), but only 17.8% of participants currently had a job. Most of them thought that they had sufficient income (78.8%). Average scores of the health status and oral health status were 3.26 (SD = 0.66, range: 1~5) and 3.28 (SD = 0.63, range: 1~5), respectively. The majority of participants had chronic diseases (74.4%) and had missing teeth (67.4%). There were 51.4% participants with visual problems and 20.9% with hearing problems. The average frequency of attending leisure activities per month was 1.09 (SD = 2.42). Average scores for the BI (ADLs), Lawton IADLS, SPMSQ (cognitive function), MSPSS (social support), and GDS-SF (depression) were 93.35 (SD = 9.51), 5.67 (SD = 2.46), 8.34 (SD = 1.84), 67.99 (SD = 10.98), and 6.46 (SD = 3.01), respectively (Table 1). 

### 3.2. Prevalence of Loneliness

There were 870 (64.0%) older adults who experienced loneliness and 490 (36.0%) older adults who did not. For each response in the single-item question, 12.7% of older adults answered “always”, 51.2% answered “sometimes”, 19% answered “seldom”, and 17.1% answered “never.”

### 3.3. Univariate Logistic Regression

Univariate logistic regression was used to examine the association between independent variables and loneliness. The results showed that age, gender, living status, number of children, health status, oral health status, chronic diseases, teeth problems, hearing problems, number of times attending leisure activities, ADLs, IADLs, cognitive function, and depression were significantly associated with loneliness among community-dwelling older adults in Indonesia (Table 2).

### 3.4. Multivariate Logistic Regression

Before conducting the multivariate logistic regression analysis, multicollinearity was checked. The variance inflation factor (VIF) was between 1.024 and 1.624. These values indicated that multicollinearity did not occur. Significant variables found in the univariate logistic regression analysis were entered into the multivariate logistic regression analysis. Results showed that gender (adjusted odds ratio (AOR): 1.43, 95% CI: 1.08~1.88, *p* = 0.011), living status (AOR: 0.32, 95% CI: 0.16~0.66, *p* = 0.002), number of children (AOR: 0.89, 95% CI: 0.84~0.94, *p* < 0.001), health status (AOR: 0.78, 95% CI: 0.63~0.97, *p* = 0.027), oral health status (AOR: 0.79, 95% CI: 0.63~0.98, *p* = 0.030), chronic diseases (AOR: 1.92, 95% CI: 1.41~2.62, *p* < 0.001), hearing problems (AOR: 0.67, 95% CI: 0.48~0.95, *p* = 0.023), cognitive function (AOR: 0.85, 95% CI: 0.78~0.93, *p* < 0.001), and depression (AOR: 1.29, 95% CI: 1.24~1.35, *p* < 0.001) were predictors of loneliness in older adults in Indonesia (Table 2). 

## 4. Discussion

This study found that the prevalence of loneliness among community-dwelling older adults in Indonesia was very high (64.0%). Compared to studies conducted in Western and Eastern countries [4,6,7,9,49], the findings of the present study revealed a serious problem that should alert government officers, social workers, and healthcare professionals to the issue of loneliness among the older population in Indonesia. Appropriate strategies should be developed immediately to eliminate loneliness experienced by older adults, such as intergenerational programs [50] and life-review therapy [51]. Moreover, qualitative research can be conducted to understand the context of the high prevalence of loneliness among older adults in the community in the future.

In the current study, older female adults were more likely to experience loneliness than older male adults. A previous systematic review reported similar findings [21]. A possible reason might be that older female adults have a longer life expectancy, so they might become widowed and have to face declining health and physical functions. Therefore, women were more prone to experience loneliness than men [52]. In contrast, a meta-analysis study found that levels of loneliness showed no difference between men and women [53]. However, interventions could be used for alleviating loneliness among female older adults, such as a friendship enrichment program [54].

Our study revealed that older adults who had fewer children experienced greater loneliness. In line with available empirical evidence, a study from Europe found that older adults who had no children also felt greater loneliness [23]. Older people being able to give affection to and receive affection from their children face a reduced extent of loneliness [55]. In contrast, some studies found that the number of children or having children did not have a significant relationship with loneliness [23,56]. In Indonesian culture, a popular proverb, “*banyak anak, banyak rezeki*”, means that the more children you have, the richer you become. Having children is a source of peace and increases one’s social status in Indonesia [57]. This might explain why older adults with fewer children experience greater loneliness.

The current study showed that older adults who lived alone were likely to experience less loneliness as compared to those living with their families. This result was similar to the findings of the previous studies by Arslantas et al. [56] and Devkota et al. [10]. However, the majority of studies indicated that people who live alone are more likely to experience loneliness than those who live with family members [7,24,31]. A possible explanation might be that the family members are not aware of the older adults’ feelings and they pay little attention to them. Moreover, older adults in Indonesia are strongly influenced by the culture of their communities. Southeast Sulawesi is one of the largest islands of Indonesia, with a large population that has a specific cultural heritage, defined as “*gotong royong*”, where neighbors regularly visit older adults to provide help on every religious occasion and for family gatherings. This kind of culture might help older adults who live alone to still feel that they are valued, can express their concerns, and are not isolated. In addition, the previous literature has revealed that living alone and loneliness are different concepts [58]. Older adults who lived alone did not indicate that they might feel lonely.

We found that older adults with poor health status and more chronic diseases experienced more loneliness. The previous literature also noted that an increase in the number of diseases was associated with greater chances of developing and experiencing loneliness [30]. A possible reason is that older adults who have better health conditions are more independent, so they do not need to depend on other people in their daily lives. In addition, they may have better connections with society. In addition to the health status, the current study also showed that older adults who had a poor oral health status had higher odds of experiencing loneliness. Several studies have reported similar results [25,59]. Poor oral health among older adults affects their quality of life and well-being [25]. Oral diseases, such as decay, gingival bleeding, calculus, caries, tooth loss, and not using dentures, isolated them from interacting with other people [26,60].

Surprisingly, our study found that older adults with hearing problems were less likely to experience loneliness than older adults without hearing problems. This finding was in contrast with several studies that showed that individuals with greater hearing loss were significantly associated with greater loneliness [29,61,62]. However, Picou and Buono [63] revealed no significant relationship between hearing loss and loneliness. A previous study conducted by Husain, Carpenter-Thompson, and Schmidt [64] suggested that hearing loss might slow reaction times to effective stimuli. A person’s hearing impairment could have an effect on their ability to understand or recognize emotional stimuli such as pleasant, unpleasant, exciting, or calming emotions. Therefore, the possible reasons for older people with hearing problems feeling less lonely might be that they cannot clearly hear about conflicts, complaints, or emotionally evocative non-speech sounds in their surroundings, so their mood might not easily be influenced by these negative words or sounds. In addition, when people have hearing problems, they are used to being surrounded only by their own voices, so they might get used to being alone. It is suggested that the number of years of hearing impairment among older adults needs to be collected for advanced analysis in the future.

This study found that older adults with poor cognitive function had greater odds of loneliness. A systematic review and meta-analysis study also had a similar finding, which was that there was a positive relationship between loneliness and an increased risk of dementia [18]. Moreover, the current study also revealed that depression was significantly associated with loneliness, which echoes previous studies [31,65]. However, a 12-year cohort study concluded that poorer baseline cognition function was significantly associated with greater odds of loneliness over time, but after adjusting for baseline depression, there was no significant relationship between cognitive function and loneliness [66]. It is suggested that more studies are necessary to examine the relations among loneliness, depression, and cognitive function in older adults in the future.

There are some limitations to the current study. First, because a cross-sectional study design was used in this study, causal relationships between independent variables and loneliness could not be identified. Second, the study aimed to understand community-dwelling older adults’ loneliness, so the results cannot be generalized to older people who live in nursing homes or hospitals. Third, the study was only conducted in Kendari City, so the findings cannot be generalized to other areas in Southeast Sulawesi or in Indonesia.

## 5. Conclusions

This study found that loneliness is a serious problem among the older population in Indonesia. Healthcare professionals and social workers should pay immediate attention to older adults. Assessment of loneliness in older patients or clients is suggested to be conducted on a routine basis during practice in order to ensure early detection of this problem, especially in those who are female, live with family, have fewer children, have a poor health status, have more chronic diseases, have a poor oral status, have no hearing problems, have poor cognitive function, and have depression. Moreover, appropriate strategies to relieve or prevent loneliness should be developed in the near future.

## Figures and Tables

**Table 1:** Demographics and characteristics of participants (*N* = 1360).

Variable	*n* (%)	Mean (SD)	Range
			Min–Max
Age (years)		66.28 (±6.39)	60~100
Gender			
Male	549 (40.4)		
Female	811 (59.6)		
Education			
Literate	1172 (86.2)		
Illiterate	188 (13.8)		
Religion			
Muslim	1322 (97.2)		
Non-Muslim	38 (2.8)		
Marital status			
With partners	947 (69.6)		
Without partners	413 (30.4)		
Living status			
Living with family	1276 (93.8)		
Living alone	84 (6.2)		
Number of children		3.83 (±2.34)	0~15
Previous employment			
No	625 (46.0)		
Yes	735 (54.0)		
Current job			
Yes	242 (17.8)		
No	1118 (82.2)		
Income			
Enough	1071 (78.8)		
Not enough	289 (21.3)		
Health status		3.26 (0.66)	1~5
Oral health status		3.28 (0.63)	1~5
Chronic diseases			
No	348 (25.6)		
Yes	1012 (74.4)		
Teeth problems			
Without missing teeth	444 (32.6)		
With missing teeth	916 (67.4)		
Vision problems *			
No	660 (48.5)		
Yes	699 (51.4)		
Hearing problems			
No	1076 (79.1)		
Yes	284 (20.9)		
No. of times attending LAs/month		1.09 (±2.42)	0~30
ADLs		93.35 (±9.51)	15~100
IADLs		5.67 (±2.46)	0~8
Cognitive function (SPMSQ)		8.34 (±1.84)	3~10
Social support		67.99 (±10.98)	22~84
Depression		6.46 (±3.01)	0-14
Loneliness			
No	490 (36.0)		
Yes	870 (64.0)		

Note: LA: leisure activities; ADLs: activities of daily living; IADLs: instrumental activities of daily living; SPMSQ: Short Portable Mental Status Questionnaire. * Missing value.

**Table 2:** Univariate and multivariate logistic regression analysis of loneliness (*N* = 1360).

Variable	Univariate OR	95% CI	*p* Value	Multivariate AOR	95% CI	*p* Value
Age	1.04	1.02~1.06	<0.001	1.02	0.99~1.04	0.123
Female	1.65	1.31~2.07	<0.001	1.43	1.08~1.88	0.011
Illiterate	0.83	0.60~1.15	0.271	-	-	-
Non-Muslim	0.82	0.41~1.63	0.563	-	-	-
Without partner	1.07	0.84~1.35	0.606	-	-	-
Living alone	0.25	0.13~0.48	<0.001	0.32	0.16~0.66	0.002
No. of children	0.92	0.88~0.97	0.001	0.89	0.84~0.94	<0.001
Having previous employment	0.87	0.70~1.09	0.220	-	-	-
Without current job	0.92	0.69~1.23	0.574	-	-	-
Without enough income	1.14	0.87~1.49	0.343	-	-	-
Health status	0.75	0.64~0.89	0.001	0.78	0.63~0.97	0.027
Oral health status	0.66	0.55~0.79	<0.001	0.79	0.63~0.98	0.030
Having chronic diseases	1.74	1.33~2.27	<0.001	1.92	1.41~2.62	<0.001
With missing teeth	0.69	0.55~0.87	0.002	0.94	0.71~1.23	0.624
Having vision problems	1.20	0.96~1.50	0.102	-	-	-
Having hearing problems	0.56	0.42~0.75	<0.001	0.67	0.48~0.95	0.023
No. of times attending LAs/month	0.92	0.87~0.97	0.001	0.96	0.91~1.00	0.074
ADLs	0.97	0.96~0.99	<0.001	0.99	0.98~1.01	0.462
IADLs	0.88	0.84~0.93	<0.001	0.99	0.93~1.06	0.832
Cognitive function (SPMSQ)	0.86	0.81~0.92	<0.001	0.85	0.78~0.93	<0.001
Social support	1.01	0.99~1.02	0.185	-	-	-
Depression	1.27	1.22~1.33	<0.001	1.29	1.24~1.35	<0.001

Note: 1. OR, odds ratio; AOR, adjusted odds ratio; CI, confidence interval; ADLs, activities of daily living; IADLs, instrumental activities of daily living; SPMSQ, Short Portable Mental Status Questionnaire. 2. Significant variables found in the univariate logistic regression analysis were entered into the multivariate logistic regression analysis, including age, gender, living status, number of children, health status, oral health status, chronic diseases, teeth problems, hearing problems, number of times attending LAs/month, ADLs, IADLs, cognitive function, and depression.

## Data Availability

The datasets used and/or analyzed during the current study are available from the corresponding author upon reasonable request.
